# Professional prehospital clinicians’ experiences of ethical challenges associated with the collaboration with organised voluntary first responders: a qualitative study

**DOI:** 10.1186/s13049-023-01147-0

**Published:** 2023-11-14

**Authors:** Oliver Beierholm Sørensen, Louise Milling, Eva Laerkner, Søren Mikkelsen, Henriette Bruun

**Affiliations:** 1 Department of Clinical Research, University of Southern Denmark, 5000 Odense, Denmark; 2 The Prehospital Research Unit, Region of Southern Denmark, Odense University Hospital, 5000 Odense, Denmark; 3 Department of Regional Health Research, University of Southern Denmark, 5000 Odense, Denmark; 4 Department of Cardiology, Nord Zealand Hospital, 3400 Hillerød, Denmark; 5 Department of Anaesthesiology and Intensive Care Medicine, Odense University Hospital, 5000 Odense, Denmark; 6 Department of Clinical Research, Research Unit in Anesthesiology, University of Southern Denmark, 5000 Odense, Denmark; 7 Psychiatric Department Middelfart, Mental Health Services in the Region of Southern Denmark, 5500 Middelfart, Denmark

**Keywords:** Ethical challenges, Emergency medical technicians, Prehospital physicians, Volunteer first responders, Community first responders

## Abstract

**Background:**

Volunteer First Responders are used worldwide. In the Region of Southern Denmark, two types of programs have been established. One of these programs consists of voluntary responders without any requirements of education or training who are summoned to prehospital cardiac arrests. The other type of program is established primarily in the rural areas of the region and consists of volunteers with some mandatory education in first aid. These volunteers are summoned to all urgent cases along with the ambulances. Cooperation between professional healthcare workers and nonprofessionals summoned through official channels may be challenging. This study aimed to explore prehospital clinicians’ experiences of ethical challenges in cooperation with volunteer first responders.

**Methods:**

We conducted 16 semi-structured interviews at four different ambulance stations in the Region of Southern Denmark. Five emergency physicians and 11 emergency medical technicians/paramedics were interviewed. The interviews were transcribed, and the data were analysed using systematic text condensation.

**Results:**

The study's 16 interviews resulted in the identification of some specific categories that challenged the cooperation between the two parties. We identified three main categories: 1. Beneficence, the act of doing good, 2. The risk of harming patients’ autonomy 3. Non-maleficence, which is the obligation not to inflict harm on others.

**Conclusion:**

This study provides an in-depth insight into the ethical challenges between prehospital clinicians and voluntary first responders from the perspective of the prehospital clinicians. Both programs are considered to have value but only when treating patients with cardiac arrest. Our study highlights potential areas of improvement in the two Danish voluntary programs in their current form.

## Introduction

Volunteer First Responders (VFRs) are used throughout the world [1–3]. There is an international consensus that these first responder programs save lives [4]. First responder programs are associated with increased odds for OHCA patients to receive bystander defibrillation and cardiopulmonary resuscitation (CPR) [5–7] and may increase 30-day survival [5, 6, 8, 9]. In Denmark, the Emergency Medical Service (EMS) is alerted through a national emergency number 1-1-2. The call is directed to a healthcare professional at the emergency medical dispatch centre (EMDC) [10]. The EMDC can dispatch an ambulance, an ambulance and a paramedic, or an ambulance and a Mobile Emergency Care Unit (MECU). The MECU is manned by an anaesthesiologist [11]. In addition to the ambulance and the MECU, volunteer first responders are activated from the EMDC in case of suspicion of OHCA or critical illness.

Two different types of VFR programs have been established in Denmark, the HeartRunner program and the Community First Responder program. The HeartRunner program was established for out-of-hospital cardiac arrest (OHCA) only. It was initiated in 2017 and was fully implemented in all Danish regions in 2020 [12]. HeartRunners are volunteers over the age of 18 years who are summoned through an app on their phones. There is no requirement regarding CPR training for the volunteers signing up for the program. The EMDC sends a message through the app to all HeartRunners within 5000 m of the patient with OHCA [13]. The alarm is sent to the 20 HeartRunners closest to the suspected OHCA. The first four who accept the alarm are forwarded to an Automated Extern Defibrillator and then to the scene of the OHCA. The fifth is sent directly to the address to provide CPR. This procedure is repeated until all HeartRunners who accept the alarm have an assignment [5, 13]. HeartRunners are dispatched by the Emergency Medical Dispatch Centre (EMDC) in all cases of suspected cardiac arrest in the entire region, regardless of whether an incident occurs in rural or urban areas.

The Community First Responder programs, on the other hand, only operate in the rural parts of the Region of Southern Denmark [14]. The responders within these programs are dispatched in all cases where an ambulance is dispatched with lights and sirens. Community First Responder volunteers are thus dispatched not only for OHCA but also for several acute medical emergencies such as respiratory problems, stroke, bleeding, sprains, and minor fractures. Additionally, the volunteers have a delegation to administer different drugs, including intramuscular adrenaline, oral glucose gel, and oral acetylsalicylic acid [15].

In a Danish cross-sectional questionnaire study, Danish emergency physicians viewed VFRs as a valuable aid to OHCA when they assisted the physicians with CPR or carried their equipment. However, the study highlighted challenges in the collaboration between VFRs and prehospital physicians, such as citizen responders being mistaken for relatives, citizen responders causing time-consuming communication, or causing crowding problems during resuscitation [16].

Ethical challenges may occur when "*there is doubt, uncertainty or disagreement about what is morally good or right*" [17]. Ethical challenges and conflicts have been demonstrated elsewhere in prehospital emergency medicine [18, 19]. Integrating nonprofessional volunteers into a professional prehospital program could potentially also lead to ethical challenges between the ambulance staff and the first responders. Even though VFR programs are widespread worldwide, the possible existence of such ethical challenges between prehospital clinicians and VFRs from the prehospital clinicians' perspective is as yet unexplored.

This study aims to explore prehospital clinicians’ (prehospital physicians, paramedics, and emergency medical technicians) experiences of ethical challenges in the cooperation with voluntary first responders.

## Method

### Study design

We applied a phenomenological approach [20]. We chose individual semi-structured interviews to explore prehospital clinicians’ experiences because this method is suitable for gaining insight into subjective perspectives [21]. We followed the Consolidate Criteria for Reporting Qualitative Research to report our study [22].

### Setting

We conducted the interviews at four different ambulance stations, two urban and two rural, in the Region of Southern Denmark. The catchment areas of the four stations differ demographically [23]. See Table 1.

**Table 1:** Interviewed prehospital clinicians and their work location

Demographics		Prehospital emergency personnel	
Ambulance Station	Catchment area^a^	MECU^b^ anaesthesiologists	EMT^c^ and Paramedic^d^
Odense C	205,000 citizens / 305 km^2^	2 males, 1 female	4 males, 1 female
Svendborg	60,000 citizens / 417 km^2^	1 male, 1 female	2 males, 0 female
Langeland	12,500 citizens / 284 km^2^	0 male, 0 female	2 males, 0 female
Otterup	30,000 citizens / 452 km^2^	0 male, 0 female	2 males, 0 female
Total	N/A	3 males, 2 females	10 males, 1 female

^a^Citizens [23]

^b^Mobile Emergency Care Unit

^c^Emergency Medical Technician

^d^Paramedic

### Participants

Five anaesthesiologists from two MECUs and 11 paramedics or emergency medical technicians (EMTs) from all four ambulance stations were included in our study. The participants were purposefully selected as they all had practical experience in collaborating with VFRs. We strived for variation in the prehospital background and gender of the respondents to enhance information power in the study [24]. See Table 1.

### Procedures

The primary author (OS) participated as a medical student in the prehospital work in all stations and conducted all interviews between August and October 2022.

The study was introduced at all four ambulance stations before the participants were recruited. Before the participants were recruited, the research team reflected on pre-existing opinions and previous interactions with the EMS and VFRs that perhaps conflicted with the aim of this paper. We thus deliberately excluded participants with already known prejudices or strong opinions about the research question.

All participants were informed both in writing and verbally that their participation in the study was voluntary and that they, at any time, could withdraw their consent to participate without penalty. Written consent statements were collected. The interviews were conducted face-to-face in an undisturbed room at the participant’s respective ambulance station during work hours. The semi-structured interviews were based on an interview guide. This interview guide was created using the framework of five phases described by Kallio et al. [25] (See Appendix 1 for a translated interview guide). All interviews were audio recorded and transcribed verbatim by an experienced medical secretary. The transcripts were not returned to participants for comments or corrections. The interviews were anonymised. Data were stored in an encrypted SharePoint site hosted by the Region of Southern Denmark.

### Ethics

According to Danish Legislation, interview studies based on informed consent do not require the approval of a scientific ethical committee in Denmark.

### Data analysis

The first author (OS) coded the data closely supervised by two senior authors (LM and HB). All data were analysed in close collaboration with all of the research team.

The method used to analyse the transcriptions was the Systematic Text Condensation method by Malterud [26]. The analysis consisted of four steps (1) *Total impression* – the establishing an overview of the data and identification of preliminary categories for further analysis; (2) *Identify and sort meaning units* – the sorting of passages related to the preliminary categories into meaning units to further reflect upon commonalities and differences in categories; (3) *Condensation* – the condensation of meaning units in each category by renaming and redefining codes through discussion between the research group using a framework of the Principles of Biomedical Ethics [28]; 4) *Synthesising* – here, data were conceptualised, and it was ensured that the synthesising results still reflected the original context. The condensates and quotations from step three were used as data in the analytic text. These steps were repeated multiple times to ensure credibility and to reflect upon the results. NVivo Pro 20 (QSR International Pty Ltd. New NVivo, 2020) was used in the coding and analysis process. Finally, the research team discussed and addressed the robustness of the final categories to ensure scientific coherence in the study. An example of the analytical process is shown in Table 2.

**Table 2:** Examples of the Systematic Text Condensation process

Text Example	Sub category	Category
“I appreciate being able to be released from resuscitation and instead talk to the relatives, thereby getting an overview of the patient's disease history”- Prehospital clinician 1	An extra set of hands in OHCAs	Beneficence, the act of doing good
“They more or less force their way into people's homes completely disrespectfully and with no regard to anything else”- Prehospital clinician 15	Privacy and the potential infringement of same	Risk of harming patient´s autonomy
“I have experienced community first responders giving oral acetylsalicylic acid. Once, it turned out that the patient had a stomach ulcer. Here it might not be wise to give acetylsalicylic acid. A second time, we discovered that the acetylsalicylic acid that had been given was one-year past its expiration date”- Prehospital clinician 9	Erroneous medicine administration	Non-maleficence, or the obligation not to inflict harm on others

## Results

Sixteen interviews with five prehospital anaesthesiologists and 11 paramedics or emergency medical technicians were carried out. The interviews varied from 7:51 to 34:18 min.

### Beneficence, the act of doing good

Most prehospital clinicians believed the programs could make a noticeable difference for the individual patient in the event of cardiac arrest in cases with longer ambulance response times. However, some prehospital clinicians mainly considered Community First Responders as a "comfort group*"* holding the patients' hands until EMS arrival rather than executing an actual evidence-based treatment in non-cardiac arrest emergencies.

“It may provide some sense of security for the relatives that people who appear to have somewhat control of the situation will arrive. It might be good in situations with a long wait for an ambulance” -Prehospital clinician 3

Overall, the prehospital clinicians described that they valued the volunteers attending cardiac arrests. It was considered good support and beneficial to have an extra set of hands available when initiating advanced resuscitation or in case of CPR-associated fatigue in the care providers. Some used the VFRs for practical assignments such as carrying utensils and monitoring equipment or even for carrying the patient. The prehospital clinicians experienced a great willingness from the VRFs to help.

*“I appreciate being able to be released from resuscitation and instead talk to the relatives, thereby getting an overview of the patient's disease history” -Prehospital clinician 1*

Thus, the prehospital clinicians believed that both the HeartRunner and the Community First Responder programs have value during cardiac arrest. However, they considered HeartRunners a more significant asset because their sole purpose was the treatment of cardiac arrest patients.

### Risk of harming patient´s autonomy

Several prehospital clinicians expressed concerns for the patient's privacy and the potential infringement when volunteers were dispatched to a patient. Some prehospital clinicians expressed an assumption that patients had contacted the emergency services to receive professional help and not volunteers without a professional healthcare background.

“*Personally, I would have a problem with someone else deciding to have other people invade my home though I have not asked for it. I have summoned professionals, not volunteers” -Prehospital clinician 11*

“*They more or less force their way into people's homes, completely disrespectfully and with no regard to anything else” -Prehospital clinician 15*

Among the prehospital clinicians, the overall perception of why volunteers would join the two programs was a desire to help others. Some prehospital clinicians experienced that health professionals felt obligated to join the programs as their professional skills were well known in the local community.

*“I[f you are not part of the program I] can imagine it to be incredibly frustrating to learn of an incidence later knowing that you might have helped your neighbour if needed” -Prehospital clinician 16*

However, many prehospital clinicians believed that some volunteers signed up due to curiosity instead of altruistic reasons. Some prehospital clinicians even believed “curiosity” predominated over “doing good” for some volunteers. The participants provided numerous examples of volunteers staying at the location and refusing to withdraw once the EMS had arrived. Some prehospital clinicians referred to this type of volunteer as a "disaster tourist" for whom the tasks seemed like a hobby.

#### Risk of breach of confidentiality

The prehospital clinicians reported having experienced patients who were unaware that the EMDC dispatched neighbours and other people from the community in case of medical emergencies. Thus, some prehospital clinicians considered sending VFRs into people’s homes without consent a slippery slope. One prehospital clinician had even experienced patients addressing themselves directly to the ambulance station in some urgent cases instead of dialling the emergency distress number to avoid Community First Responders being involved. Further, the prehospital clinicians described experiencing breaches of confidentiality after completing a task. In these cases, Community First Responders had talked to the patient's neighbours about the health situation following the event, while others described Community First Responders who had questioned the prehospital clinicians closely about the further course of patients from previous tasks.

“*If you subsequently meet both HeartRunners and Community First Responders in other contexts, or if you meet them in the small communities, they will approach and ask: 'How did this and that go?” -Prehospital clinician 15*

### Non-maleficence, or the obligation not to inflict harm on others

Several prehospital clinicians were under the impression that the local population felt an unjustified sense of security due to the Community First Responder program and underlined that they considered the Community First Responders to make a difference solely in cardiac arrest cases due to a lack of skills in other medical emergencies. For example, several had experienced Community First Responders misinforming patients and relatives.

*“I do not believe the Community First Responders to have any justification. It is fraudulent to tell people that they are receiving help purely because a neighbour comes running, driving, or whatever they do. It is simply a false sense of security. It is not about people arriving quickly; it is about the right help being accessible. And as soon as possible, of course*” *-Prehospital clinician 2*

Prehospital clinicians reported numerous incidents of erroneous medicine being administered by Community First Responders. The prehospital clinicians perceived a lack of education and understanding on the subject as a reason. They described situations where acetylsalicylic acid was given in unfavourable situations, including stomach ulcers, aortic dissection, and unclarified stroke.

*“I have experienced Community First Responders giving oral acetylsalicylic acid. Once, it turned out that the patient had a stomach ulcer. Here, it might not be wise to give acetylsalicylic acid. A second time, we discovered that the acetylsalicylic acid that had been given was one-year past expiration date” -Prehospital clinician 9*

The prehospital clinicians considered Community First Responders located in rural areas with significantly increased response time as a useful solution. However, according to the prehospital clinicians, Community First Responders being dispatched inappropriately was a concern. The prehospital clinicians wished for the EMDC to only alert Community First Responders to relevant emergencies such as cardiac arrest. For instance, one prehospital clinician described that the EMDC had alerted Community First Responders to a woman with vaginal bleeding, where they could not perform any treatment and instead stood watching idly. Another questioned the usability of Community First Responders if they do not make any difference to the outcome.

*“There was a child with a fever and fever cramps, and then a Community First Responder arrived. What is he going to do there? That is stupidity” -Prehospital clinician 16*

Several prehospital clinicians mentioned Community First Responders as a disadvantage more than a factual medically relevant help. However, considering the abundance of tasks related to the concept of “a spare set of hands”, many believed that the Community First Responder program has a justification in its current form.

*“So, of course, we can ask them to carry things and hold a door and stuff like that. But you do not have to have such an emergency program for holding doors”* -*Prehospital clinician 10*

#### Risk of interference with the treatment performed by EMS

There were reports of situations where the prehospital clinicians had felt a competitive element from the Community First Responders. The prehospital clinicians experienced that some Community First Responders perceived it to be a race with the ambulance to get to the patient first, even though they might not have the opportunity to start treatment. They described that this behaviour had resulted in Community First Responders getting in the way of the ambulances. Prehospital clinicians described finding it annoying when Community First Responders attempted to hand over the patient due to the often irrelevant information in the handover.

*“They think they know so much but do not really know anything. Nevertheless, they want to tell us everything they know when we arrive, even though they have only been there for a minute and do not know anything. It is kind of annoying to stand there and be like: ‘That is fine” -Prehospital clinician 6*

Though most prehospital clinicians considered HeartRunners as significant assets because attending to cardiac arrest was their sole purpose, some described HeartRunners to be a disadvantage in their work. The prehospital clinicians noted being frustrated as HeartRunners at times would get in the way, ask many time-consuming questions, and take on tasks not within their area of responsibility. For instance, one prehospital clinician had experienced a HeartRunner calling the relatives of a deceased patient and informing them of their family member's passing. The prehospital clinicians described having to spend valuable time away from the patients while diverting superfluous VFRs away from the scene. Thus, the prehospital clinicians noted that HeartRunners could create new problems. According to the prehospital clinicians, these situations arose due to a lack of situational awareness from HeartRunners. The prehospital clinicians noted the primary challenges with HeartRunners were their behaviour and attitude, not their resuscitation skills.

*“I once declared a patient dead. [In the meantime] the HeartRunners nearby high-fived and were like, "Great job" to each other. And then, at the same time, there were relatives in the room next door. The HeartRunners started to loudly "debrief" each other, and I ended up telling them to shut up and go outside” -Prehospital clinician 11*

The prehospital clinicians described several experiences where VFRs had expressed their opinion concerning the treatment and even questioned the treatment initiated by the prehospital clinicians. These were both VFRs with healthcare backgrounds as well as laypersons. The prehospital clinicians experienced that the volunteers considered they had a say in the treatment because the EMDC had sent them.

*“Sometimes, the volunteers are a crowd of people from the healthcare sector standing there. They all give their opinion on the matter. We just had to show them off. It certainly did not suit them” -Prehospital clinician 2*

On several occasions, the prehospital clinicians described professional insecurity and intimidation when the VFRs did not want to hand the patient over.

*“But I just felt like I almost had to beg to be allowed to take over my patient”* *-Prehospital clinician 3*

The amount of VFRs sent in case of cardiac arrest was considered too extensive. The prehospital clinicians experienced difficulties in physically approaching the patient upon arrival because of the many VFRs who were in the way. Several prehospital clinicians had experienced 10–12 HeartRunners at the scene with some HeartRunners arriving by car and parking inconveniently and blocking the roads. Problems due to what prehospital clinicians perceived as an excessive amount of VFRs were described more often with the HeartRunner program than with the Community First Responder program.

*“I had to give up driving the ambulance onto a cul-de-sac because it was simply filled up with cars. It was just as if there was going to be a concert or something” -Prehospital clinician 2*

Prehospital clinicians stated that HeartRunners continued to arrive in large numbers after arriving at the patient, even though professional help had already arrived on site. Despite fewer volunteers being alerted in the Community First Responder program, the prehospital clinicians still reported issues distinguishing Community First Responders from the patient's relatives.

*“They are pouring in, and of course, it is fine if there is something they can do for us and help us. However, often, when there already are a few of us at the address, it can then be too much” -Prehospital clinician 14*

An issue mentioned by several prehospital clinicians was how VFRs transported themselves to the patient. Several prehospital clinicians described situations where they almost raced against the VFRs who exceeded the speed limits on their way to the patients. A concern of the prehospital clinicians was that accidents could occur because of the VFRs' eagerness to get to the patient as quickly as possible.

*“It is very unfortunate that you are driving an EMS vehicle with approximately 300 horsepower, and then you have a Community First Responder in a private car tailgating you through a village at a very high speed” -Prehospital clinician 15*

Several prehospital clinicians reported that they not only had an eye on the regular traffic but also on the inappropriate behaviour they believed VFRs had in traffic. This occasionally resulted in the prehospital clinicians having to slow down and even stop completely to avoid colliding with VFRs.

*“I experience volunteers running or cycling across the road, and suddenly, they are in the middle of the road because they are too eager to arrive as either a HeartRunner or a Community First Responder” -Prehospital clinician 14*

## Discussion

This study investigated the ethical challenges and problems in the collaboration between VFRs and EMS professionals as described by prehospital clinicians.

### Ethical principles

The four moral principles in biomedical ethics are described as "(1) Respect for autonomy (the obligation to respect the decision-making capacities of autonomous persons), Non-maleficence (the obligation to avoid causing harm), (3) Beneficence (obligations to provide benefits and to balance benefits against risks) and (4) Justice (obligations of fairness in the distribution of benefits and risks)" [27, 28]. These principles are universally applicable and should function as guidelines for professional ethics [27].

In this context, we identified three overarching categories: Ethical challenges concerning the patients, Ethical challenges concerning collaboration with Volunteer First Responders, and Ethical challenges concerning EMS personnel.

### Ethical challenges concerning the patients

The prehospital clinicians in our study found the missing patient consent to the presence of VFRs problematic. The principles of biomedical ethics state the necessity of patients receiving relevant information to understand and assess possible consequences [27]. The patients and health professionals must share a mutual understanding of the terms of authorisation before proceeding with any actions, and the patients' autonomous wishes must be respected [27]. While the prehospital clinicians were concerned about the ethical dilemmas surrounding volunteers entering patients' homes, the European Resuscitation Council values the potential of saving lives higher than the ethical dilemma of the breach of privacy [29].

A study by Dainty et al. [30] concluded that approximately 85% of the study population had no problem receiving CPR from volunteers in their private homes. To our knowledge, no studies have investigated patients' attitudes toward receiving help from volunteers in cases other than OHCA. Breaching of the patient´s privacy by VFRs entering a patient´s home in other cases than OHCA, for example in a case of vaginal bleeding, was the main concern of the prehospital clinicians.

The concept of training and skills among VFRs was also highlighted. As in our study, Dainty et al. [30] reported concern among the study population about receiving help from volunteers who lack training and skills. This finding is relevant to the Danish setting, where there is no requirement for CPR training in the HeartRunner program. The Community First Responder program, on the other hand, requires a first aid course, but our prehospital clinicians still reported a lack of skills in cases other than OHCA.

Beauchamp and Childress refer to the individual right to give other people access to personal information. Even though others may get to know that someone is sick, it will violate the privacy of the person concerned if details about the disease are exposed [27]. This topic was a concern to our prehospital clinicians who reported experiences of volunteers disclosing personal and sensitive information to others, thereby violating their duty of confidentiality. A study by Nabecker et al. [31] described volunteers experiencing difficulties with questions from community citizens after VFR tasks. Confidentiality is a "prima facie" in ethics [27]. The potential for breaches of confidentiality should therefore be addressed in future research.

In our study, prehospital clinicians believed Community First Responders were summoned to many tasks where the Community First Responders did not contribute to appropriate treatment but instead stood in the way. From 2012 TO 2017, only 112 out of 2688 activations of Community First Responders in a small defined rural area within our region concerned cardiac arrests [8]. As the responders in this study primarily found participation by VFRs valuable in cardiac arrest, this may explain our study participants’ attitudes towards the varied use of Community First Responders.

### Ethical challenges concerning collaboration with volunteer first responders

The principle of respect for autonomy includes the right to decide what will happen to oneself [27]*.* The prehospital clinicians in our study experienced that VFRs only sometimes respected when patients rejected their help, and that VFRs considered it their right to help because they were dispatched by the EMDC.

The prehospital clinicians experienced that the motivation for participating in the VFR programs was primarily altruistic. These motivational factors of VFRs are known from other studies [32–34]. However, some VFRs may fail to act altruistically because of their self-interest motivations [27, 35]. It has been reported that some volunteers participate as first responders for the thrill and to obtain an "adrenaline rush". This group includes not only laypersons but also general practitioners who wish to receive an "adrenaline rush" [36] as a VFR. Voluntary laypersons describe other voluntary laypersons as "blue-light junkies" [2]. Our findings support this with our prehospital clinicians referring to these VFRs as "disaster tourists".

According to our study, healthcare professionals might feel obligated to join first responder programs. The European Resuscitation Council states that bystander CPR is a voluntary act with no moral or legal accountability [29]. As such, no one is morally obligated to join voluntary programs.

VFRs could, in some situations, act unintentionally careless according to the prehospital clinicians in our study. The prehospital clinicians speculated that this could end up imposing a risk of harm. One example was exceeding the speed limit on the way to the scene; another example was incorrect administration of medicine to patients.

Allowing volunteers without formal medical education to administer drugs can be debated "*Professional malpractice is an instance of negligence that involves not following professional standards of care. These standards require proper training, skills, and diligence."* [27]. Volunteers are not health professionals and they sometimes deviate from the usual indications when administering drugs. If the volunteers cannot be held accountable for erroneous administration of medicine, it could be argued that they should not be allowed to administrate these types of medicine. The official Community First Responder manual [15] states that Community First Responders act on behalf of the Region of Southern Denmark and are a part of the prehospital setup. Therefore, they should have the same professional care standards as the EMS. Our study’s prehospital clinicians suggested that Community First Responders should only be alerted and sent out to OHCA because of their lack of skills regarding other medical emergencies. This was a major topic among the prehospital clinicians in our study as they experienced Community First Responders as passive and helpless in emergencies.

A study by Nabecker et al. [31] reported that volunteers felt anxious and helpless in cases other than OHCA because they lack the competence to act. Phung et al. [34] reported anxiety and stress among Community First Responders when they were first on the scene.

In our study, prehospital clinicians reported Community First Responders standing passively by in most non-OHCA situations. Therefore, emotional support, education, and triage of VRFs should be improved to protect the VFRs and to protect patients against incorrect treatments performed by the VFRs.

Phung et al. [34] elucidate the feeling of being an asset in providing essential information to the ambulance staff among the Community First Responders. The Community First Responders act as if they gather essential clinical information for the EMS. However, in our study, prehospital clinicians describe the information as a waste of time as they often must collect the information again themselves.

Some studies [16, 37] share our findings regarding the advantages of volunteers in the case of OHCA. These studies state that volunteers and laypersons, in general, are considered an asset in this situation.

### Ethical challenges concerning EMS personnel

The high number of VFRs attending an emergency can be challenging for prehospital clinicians. A survey study by Jellestad et al. [16] demonstrated that 20% of MECU physicians experience problems with HeartRunners. These problems include difficulties distinguishing the numerous volunteers from the relatives. However, 92.5% of MECU physicians considered volunteers relevant in OHCA resuscitation, and approximately 68% found the collaboration helpful. Three out of four MECU physicians used volunteers to continue CPR and to carry equipment. These findings correspond with and are nuanced by the findings in our study.

The presence of VFRs could make an already difficult situation even more stressful for prehospital clinicians [37]. This was supported by our study of prehospital clinicians. Ethical challenges in the prehospital emergency setting are not only related to the VFRs. Previous studies have identified other themes regarding situations where EMS experiences challenges and conflicts concerning ethical considerations such as caring for patients, the professional role and self-identity, and external collaboration [18, 19, 38].

Milling et al. showed that EMS personnel are potentially affected by bystanders in cases of OHCA [38]. Bystanders could potentially influence prehospital clinicians to continue CPR and make them feel obligated to continue CPR if the bystanders already had initiated resuscitation to motivate bystander CPR in future OHCAs [38]. The study reports that prehospital clinicians may feel frustrated because of bystanders' unrealistic expectations [38]. Therefore, using VFRs may add a challenging dimension to the work of EMS professionals. This correlates with the results of our study.

Future studies should investigate whether patients and relatives feel well informed about VFRs or if they experience a violation of the principle of respect for autonomy, or even feel unsafe with VFRs present. It is necessary to explore the value of VRFs in cases other than OHCAs to evaluate their influence on patient survival rates in non-OHCAs.

### Strengths and limitations

Our study strengths include the first author's active involvement in the daily prehospital work, thereby achieving a deep insight into experiences with first responders. Furthermore, no respondents declined to participate in our study. This suggests a desire to express views and experiences.

Our study has one main limitation. Our results reflect the attitude of the interviewed EMS personnel but might not be generalisable to other settings. However, other studies have described similar challenges associated with professional/volunteer collaboration, although these mainly explore the themes or categories from the volunteers' point of view [31, 32, 39].

## Conclusion

This study provides in-depth insight into the ethical challenges and tensions between prehospital clinicians and volunteer first responders from the perspective of the prehospital clinicians. Volunteer first responder programs undoubtedly are initiated out of good intentions. However, our results indicate that the volunteer first responder risk interfering with the professional emergency medical system. The prehospital clinicians thus considered that volunteer first responder programs only have value in the treatment of patients with cardiac arrest. Therefore, both to avoid harming patients and to avoid the risk of the volunteer first responders feeling helpless in situations exceeding their competencies, automatic dispatch to medical emergencies per se was considered problematic. Our study highlights a need for optimising Danish voluntary programs in their current form regarding training, information on how to act proactively and respectfully, as well as considerations about the number of volunteers dispatched. Furthermore, re-evaluation of the nature of the tasks the volunteers are dispatched to is warranted.

## Data Availability

Anonymised transcripts of the interviews are available in Danish on reasonable request.

## Appendix 1: Interview guide

**Table:**

Main questions	Followup question
What is your knowledge of the Heartrunner / community first responder program?	1. How often do you experience meeting heartrunners /community first responders through your work? 2. Are you a heartrunners /community first responder in your spare time, and how many times have you been alerted? No knowledge: Thank you for your time –––––––––––––––––––––––––––––––- • Can I get you to elaborate on your answer? • Do you have an example of your experiences?
Elaborate on your experiences with heartrunners / community first responders?	• Can I get you to elaborate on your answer? • Do you have an example of your experiences?
How do heartrunners / community first responders support you in your work?	• How do they affect your work at the site of emergency? • Is there a difference whether it is a heartrunner or a community first responder who attends? • What is your experience of their motivation? / What motivates them? • What do you think of the two programs? –––––––––––––––––––––––––––––––- • Can I get you to elaborate on your answer? • Do you have an example of how the heartrunners /community first responders support your work?
How do heartrunners / community first responder challenge you in your work?	• How do they affect your work at the site of emergency? • Is there a difference whether it is a heartrunner or a community first responder who attends? • How do you assess their CPR? • What is your experience of their motivation? / What motivates them? • What do you think of the two programs? –––––––––––––––––––––––––––––––- • Can I get you to elaborate on your answer? • Do you have an example of how the heartrunners /community first responders challenge your work?

## Abbreviations

CPR: Cardiopulmonary resuscitation
EMDC: Emergency medical dispatch centre
EMS: Emergency medical service
EMT: Emergency medical technician
MECU: Mobile emergency care unit
OHCA: Out-of-hospital cardiac arrest
VFR: Volunteer first responder
